# MicroRNA-1 Negatively Regulates Peripheral NK Cell Function via Tumor Necrosis Factor-Like Weak Inducer of Apoptosis (TWEAK) Signaling Pathways During PPRV Infection

**DOI:** 10.3389/fimmu.2019.03066

**Published:** 2020-01-23

**Authors:** Xuefeng Qi, Zhen Li, Huan Li, Ting Wang, Yanming Zhang, Jingyu Wang

**Affiliations:** College of Veterinary Medicine, Northwest A&F University, Yangling, China

**Keywords:** MicroRNA-1, TWEAK, PPRV, goat, NK cells

## Abstract

Peste des petits ruminants virus (PPRV) has emerged as a significant threat to the productivity of small ruminants worldwide. PPRV is lymphotropic in nature and induces in the hosts a transient but severe immunosuppression, especially innate immunity. However, it remains largely unknown how NK cells respond and are regulated at the earliest time points after an acute viral PPRV infection in goats. In this study, we revealed that multiple immune responses of goat peripheral NK cells were compromised during PPRV infection, including the cytolytic effector molecule expression and cytokine production. Importantly, we demonstrated that PPRV infection stimulated the expression of TWEAK, a negative regulator of cytotoxic function of NK cells, which may be involved in the suppression of cytotoxicity as well as cytokine production in infected goat NK cells. Furthermore, we found that PPRV infection induced TWEAK expression in goat NK cells involving post-transcription by suppressing miR-1, a novel negative miRNA directly targeting the TWEAK gene. Moreover, replication of virus is required for inhibition of miR-1 expression during PPRV infection, and the non-structural V protein of PPRV plays an important role in miR-1 mediated TWEAK upregulation. Additionally, we revealed that the regulation of NK cell immune responses by TWEAK is mediated by MyD88, SOCS1, and STAT3. Taken together, our results demonstrated that TWEAK may play a key role in regulating goat peripheral NK cell cytotoxicity and cytokine expression levels during PPRV infection.

## Introduction

Peste des petits ruminants virus (PPRV) is a member of the Morbillivirus family and is one of highly contagious fatal diseases known to domestic and wild small ruminants (1–3). PPRV infection usually caused severe suppression of immune responses in goat, which favors secondary infections (4–6). Although the vaccine with attenuated PPRV has been found to be highly efficacious, safe, and potent in small ruminants, it has been shown that the vaccine virus induced an early and transient lymphopenia in goats (7). Importantly, transient immune suppression to other antigens in goats immunized with PPRV vaccine was found (7). Goats are naturally more susceptible to PPRV than sheep due to the host- or virus-derived factors (1, 8). PPRV, like most morbilliviruses, has a well-established receptor-dependent lymphotropism and epitheliotropism. PPRV enters lymphoid cells through signaling lymphocyte activation molecule (SLAM), which is widely expressed on the surface of all immune cells (9–11). The differential susceptibility could one part attributed to the host immune responses, although this has not been explored in detail in ruminant species or breeds.

NK cells are lymphocytes that eliminate virus-infected cells by both direct cytolysis and the production of cytokines and chemokines (12). At early viral infection, NK cells can direct eliminated infected cells through cytolytic activity and cytokine secretion, such as IFN-γ and tumor necrosis factor alpha (TNF-α). NKG2D, the activating receptor of NK cells, is best characterized for virus and tumor elimination and recognizes ligands that are induced by cellular transformation, stress, or infection (13). It is well-known that JAK-STAT axis transduces signals of interleukin-6 (IL-6) and interferons (IFNs). Further studies revealed that signal transducer and activator of transcription 3 (STAT3) is a positive regulator of NKG2D in NK cells (14, 15). Moreover, IFN signaling can be inhibited by various viruses through targeting SOCS1, a negative regulator of the JAK/STAT pathway (16–18). Additionally, investigation of the mechanism for MyD88-dependent NK cell dysfunction revealed that the induction of cytokines, including IL-18, IL-17, and TNF-α, is largely MyD88 dependent (19).

While controversial results about NK cell percentages following measles vaccination were reported (20), natural measles infection suppress NK cell activity as compared to uninfected individuals (6). Up to now, the immune responses of ruminant NK cells in responses to PPRV infection were limited. Ovine CD16^+^CD14^−^ lymphocyte displays the phenotype and functional characteristics of NK cells (21), which provide some insight into ruminant NK cell biology. Various cytokines can modulate NK cell function, including interleukin (IL)-12, interferon (IFN)-α, interleukin (IL)-21, and transforming growth factor (TGF)-β (22). The role of TWEAK (TNF-related weak inducer of apoptosis, also known as Apo3L or TNFSF12) in regulating NK cell function during PPRV infection has not been extensively studied. TWEAK was first described as an inducer of apoptosis in transformed cell line (23). In addition to this important function, increasing evidence suggests that TWEAK also has a prominent role in the control of the innate inflammatory response as well as the transition to Th1-based adaptive immunity (24). Interestingly, Avi Ashkenazi et al. have shown that TWEAK was mainly expressed by NK cells, macrophages, and dendritic cells in response to LPS stimulation (25). However, very little is known about the role of TWEAK in defining goat NK cell function during PPRV infection.

MicroRNAs (miRNAs) have emerged as key regulators of gene expression in the past decade. Notably, many cellular miRNAs were involved in NK cell activity and function modulation (26). Our previous study has identified 316 significantly differentially expressed miRNAs from PPRV-infected goat peripheral blood mononuclear cells (PBMCs) as compared with mock control (27). We also predicted that *TWEAK* gene is regulated by several miRNAs, including novel_miR1 and chi-miR-342-5p, by Target Scan and their fold change (27).

Studies available on the induction of both type I- and type II-interferon (IFN) during PPRV infection *in vivo* or after vaccination are inconclusive (28–32). Indeed, it has been shown that PPRV infection alone was sufficient to cause the decrease of IFN-γ production and suppression of IFN-γ activation in infected cells, including Vero cells and goat fibroblasts (28, 31, 32). This implicates a role for either PPRV itself or cellular factors regulated by PPRV replication in impairing IFN-γ-producing cells and contributing to viral persistence. At early PPRV infection, NK cells are considered as the primary source of IFN-γ (28, 32). However, it remains largely unknown how NK cells respond and are regulated at the earliest time points after an acute viral PPRV infection in goats.

Here, we demonstrate that PPRV infection stimulates a rapid increase of TWEAK expression in goat NK cells at early infection, which decrease cytotoxic potential of NK cells and downregulate IFN-γ production by NK cells. Particularly, we demonstrate that TWEAK is regulated by cellular miR-1, which then contributes to NK cell phenotype and function modulation. Moreover, decreased cytotoxicity and lower miR-1 expression correlated with increased virus production during PPRV infection. Collectively, our data demonstrate that TWEAK is a significant modulator of NK cell function and that cellular miR-1 has a role in regulating TWEAK expression during PPRV infection.

## Materials and Methods

### Animals

The clinical healthy 6-months-old goats used in this study were housed in appropriate containment facilities and had *ad libitum* access to feed and water. Goats were screened for PPRV antibodies using competitive ELISA serum neutralization test kit (Yoyoung Biotech. Co., Ltd, Guangzhou, China) and showed negative.

### Cells and Virus

Blood samples from goat were collected on EDTA vacutainers (BD Biosciences). PBMCs were isolated using Histopaque-1077 (Sigma, USA) by density gradient centrifugation following the manufacturer's instructions. NK cells were then isolated by positive immunomagnetic selection as previously described (21). The purity of the isolated CD16^+^CD14^−^ NK cells were usually over 96%, assessed by flow cytometric analysis after staining with CD16-R-Phycoerythrin (PE) (clone KD1, SouthernBiotech, Birmingham, USA) and CD14^−^Tricolor (TC) mAbs (CAM36A, VMRD, Pullman, USA). The goat NK cells were maintained as previously described (21) in RPMI-1640 medium (Hyclone, Logan, UT, USA), supplemented with 60 μg/ml penicillin, 100 μg/ml streptomycin, 10% fetal calf serum (FCS, Invitrogen), and 100 U/ml recombinant human (rh) IL-2 (R&D Systems).

The PPRV vaccine strain, Nigeria 75/1, was obtained from the Lanzhou Veterinary Research Institute, Chinese Academy of Agricultural Sciences (Lanzhou, China). Virus stock was prepared by collecting the infected Vero cell supernatant when cytopathic effect (CPE) affected about 80% of the cells. The virus was harvested by three cycles of freezing and thawing and stored at −80°C and purified by banding on sucrose gradient (33). The purified virus titers were estimated by estimating 50% tissue culture infective doses (TCID_50_) using Vero cells in 96-well microtiter plate. The purified virus was tested for its infectivity in Vero cells and was used further for infection in goat NK cells.

For virus infection, goat NK cells were seeded into 96-well plates at a density of 1 × 10^5^ cells/ml and further stimulated with 500 pg/ml rh IL12 (500 pg/ml) (R&D Systems), followed by PPRV Nigeria 75/1 strain infection for the indicated time. NK cells inoculated with similarly purified preparation from triple freeze-thawed Vero cells were used as the mock-infected group. Western blot was performed using a monoclonal antibody against PPRV N protein provided by the China Animal Health and Epidemiology Center (Qingdao, China) to determine virus replication at the different time points. Three replicates of PPRV- and mock-inoculated cultures were prepared at each time point.

### Virus Titration

Virus progeny production was determined by titration as described previously (34). The viral supernatants from PPRV-infected goat NK cells were collected at the indicated time points after virus inoculation, and the 50% tissue culture infective dose (TCID_50_) was calculated by the Reed-Muench method (35).

### Flow Cytometric Analysis

Multiple labeling of surface receptors was performed on goat NK cells against the molecules: CD16 (clone KD1, SouthernBiotech, Birmingham, USA), CD14 (CAM36A, VMRD, Pullman, USA), NKp46 (CUSABIO Biotech, China), CD3 (clonal CD13-12, BioRad), and SLAM (Santa Cruz Biotechnology, Santa Cruz, CA). Intracellular perforin labeling was performed with the perforin-FITC kit (clone δG9, IgG2b, BD Biosciences, San Jose, CA) and the Cytofix/Cytoperm and Permwash solutions from BD Biosciences. Then, the samples were washed twice in cold phosphate buffered saline (PBS) and analyzed on a FACSCalibur (BD Biosciences). Cell acquisitions were performed on 2–10 × 10^4^ viable cells gated in the forward and side scatter plot for the phenotype characterization and on 1.5–2 × 10^5^ gated goat lymphocytes for the perforin assay. For evaluation of apoptosis, the cells were harvested, washed three times with PBS, centrifuged, and suspended in 500 μl of 10× binding buffer, followed by treatment with 10 μl of FITC-labeled annexin V (BD Biosciences, San Jose, CA) per sample for 10 min at room temperature. Then, the infected cells were stained with 5 μl of propidium iodide (PI) per sample for 5 min, followed by analysis on a FACSCalibur (BD Biosciences, San Jose, CA). Annexin V-positive and PI-negative cell populations in the lower right quadrant of the Annexin V vs. PI FACS plots were considered being apoptotic cells. For the detection of CD16^+^/SLAM^+^, CD16^+^/NKG2D^+^, CD16^+^/IFNG^+^, and CD16^+^/Perforin^+^, cells were incubated with CD16-R-Phycoerythrin (PE) (clone KD1, SouthernBiotech, Birmingham, USA), washed three times, and incubated with FITC-conjugated anti-SLAM antibody (Santa Cruz Biotechnology, Santa Cruz, CA), or incubated with mouse monoclonal antibodies against goat IFNG or Perforin (Abcam System, Cambridge, MA) or mouse anti-NKG2D monoclonal antibodies (BD Biosciences, San Jose, CA) followed by incubation with FITC-conjugated anti-mouse IgG antibody. Then, the cells were washed twice in cold phosphate buffered saline (PBS) and analyzed on a FACSCalibur (BD Biosciences, San Jose, CA).

### Western Blot Analysis

Protein homogenates from goat cells were extracted as previously described (36). Briefly, the cells were lysed for 20 min on ice in ice-cold lysis buffer (Roche). The lysates were centrifuged at 12,000 × *g* for 20 min at 4°C to obtain a clear lysate. The protein content of each sample was determined using the BCA Protein Assay Kit (Thermo Fisher Scientific, Waltham, MA, USA). Then, equal amounts of protein were separated on a 12% SDS-polyacrylamide gel and transferred to polyvinylidene difluoride membranes. Membranes were probed overnight at 4°C with an anti-PPRV-N monoclonal antibody provided by China Animal Health and Epidemiology Center (Qingdao, China), a rabbit polyclonal antibody against mouse IFNG (1:1,500; Abcam), a rabbit polyclonal antibody against mouse Perforin (1:2,000; Santa Cruz, CA, USA), a rabbit polyclonal antibody against mouse NKG2D (1:1,500; ABclonal, Boston, MA, USA), a rabbit polyclonal antibody against mouse TWEAK (1:1,500; ABclonal), or a rabbit polyclonal antibody against mouse TNFα (1:1,500; Abcam). For downstream signaling pathway detection, membranes were probed overnight with rabbit anti-SOCS1 (Sangon Biotech, Shanghai, China), rabbit anti-NF-κB p65 (Bioss, Beijing, China), rabbit anti-MyD88 (Bioss, Beijing, China), rabbit anti-STAT3 (Bioss, Beijing, China), or rabbit anti-pSTAT3 (Bioss, Beijing, China). Then, the bands were visualized using horseradish peroxidase (HRP)-conjugated goat anti-mouse IgG (1:15,000, Boster, Wuhan, China) or goat anti-rabbit IgG (1:20,000, Boster, Wuhan, China) prior to the ECL protocol (Amersham Biosciences, Piscataway, NJ, USA). As an internal standard, all membranes stripped with primary antibodies were reprobed with anti-GAPDH antibody (Invitrogen). Changes in protein expression were determined after normalizing the band intensity of each lane to that of GAPDH. Signal was visualized using the Konica SRX 101A developer (Konica Minolta Medical Imaging, Wayne, NJ, USA) and the Quantity One software (Bio-Rad, Mississauga, ON, Canada) was used for densitometrical analysis.

### RNA Interference

Small interfering RNAs (siRNAs) targeting TWEAK (forward, 5′-ATGCATGC-3′; reverse, 5′-ATGCATGC-3′) or control siRNA (forward, 5′-ATGCATGC-3′; reverse, 5′-ATGCATGC-3′) were designed and synthesized by RiboBio Inc. (Guangzhou, China) and then used for silencing the target genes. Briefly, purified goat NK cells were transfected with 50 nM siRNA targeting TWEAK or control siRNA by using Lipofectamine RNAiMAX according to the manufacturer's guidelines (Life Technologies, USA). After transfection, NK cells were cultured in RPMI-1640 medium supplemented with 10% FCS and rh IL-2 (R&D Systems, Abingdon, United Kingdom) for 48 h, and further stimulated with rh IL-12 (R&D Systems, Abingdon, United Kingdom) and infected with PPRV at an MOI of 1 for 24 h before the cells were harvested for Western blotting.

### Lentiviral Transduction

The TWEAK overexpression lentivirus was purchased from Bio-Transduction Lab (Wuhan, China). Goat NK cells were transduced with the indicated lentiviral stocks. Viral titer was performed at 100:1 for NK cells transduction with 4 μg/ml polybrene (Sigma-Aldrich, MO, USA). Successful expression of TWEAK protein was determined by Western blot analysis.

### miRNA Target Gene Prediction and Plasmid Construction

The bioinformatics tools RNAhybrid (37) and TargetScan (38, 39) were utilized to determine potential target genes of differential expressed miRNA. Each 3′UTR and mutant 3′UTR of the potential target genes listed in Figure 4B were synthesized by Sangon Biotech (Shanghai, China) and cloned into psiCHECK-2 (Promega, Madison, USA) using the Notl and Xhol restriction sites.

### Dual-Luciferase Reporter Assay

HEK293 cells were transfected with 10 ng each of psiCHECK-2 reporter plasmids along with 15 pmol of the miR-218 mimic or an identical amount of the negative control with Lipofectamine 2000 (Invitrogen). After 48 h, the cells were lysed, and the firefly and Renilla luciferase activities were measured with the Dual-Luciferase Reporter Assay System Kit (Promega, Madison, USA) according to the manufacturer's protocol. Each fragment containing the putative miRNA-binding sites was cloned in psiCHECK-2 immediately downstream of the gene encoding Renilla luciferase by the Protein Expression Laboratory (SAIC, Frederick, MD). The results are presented as the ratio of Renilla luciferase activity to firefly luciferase activity. Each transfection was performed at least three times and was assayed in triplicate.

### RNA Isolation and Real-Time PCR Analysis

Total RNA was extracted from goat NK cells using TRIzol reagent (Invitrogen, Waltham, MA, USA) according to the manufacturer's instructions. RNA was then reversed using Superscript III (Invitrogen) and random primers (Invitrogen). Real-time quantitative PCR was carried out using an ABI 7500 System (Applied Biosystems, Warrington, UK) and Power SYBR Green PCR Master Mix (Applied Biosystems, Warrington, UK). The sequences of the primers used were as follows: *TWEAK*, 5′-CGCTGTTTGCCCAGGAGCCTT-3′ (forward), 5′-CGCCCGTGGTTTCTGGCCTT-3′ (reverse); *GAPDH*, 5′-AATGAAAGGGCCATCACCATC-3′ (forward), 5′-GTGGTTCACGCCCATCACA-3′ (reverse). The PCR cycling conditions were 20 s at 95°C, followed by 40 cycles of 3 s at 95°C and 30 s at 60°C. Expression of *GAPDH* gene was used to normalize cDNA levels for differences in total cDNA levels in the samples. Then, the Ct (*d*) was used to calculate the fold difference in copy number using the formula *f* = 2^(−d)^, where *f* = the fold difference in the expression of a specific gene and *d* = the difference in the *Ct* values between the compared sources of mRNA (corrected for differences in the GAPDH levels). We normalized each sample to control cell sample #1. Melt curves were performed to confirm the purity of the amplified products.

For detection of miR-218, total RNA was reverse transcribed and quantitative real-time RT-PCR analysis was performed using Bulge-loop™ miRNA qRT-PCR Primer Sets (one RT primer and a pair of qPCR primers for each set). The primers for miR-218 and internal standard 5S snRNA are designed by RiboBio Inc. (Guangzhou, China) and the sequences are covered by a patent. Briefly, 2 μl of cDNA was added to 10 μl of the 2 × SYBR green PCR master mix with 0.2 μl of Taq polymerase enzyme (RiboBio, China), 200 nM of each primer, and ddH_2_O to a final volume of 20 μl. The reactions were amplified for 2 s at 95°C and 20 s at 60°C for 40 cycles. The thermal denaturation protocol was run at the end of the PCR to determine the number of products that were presented in the reaction mix. Reactions were typically run in duplicate. Micro RNA relative expression quantity was detected and calculated using relative quantitative standard curve method.

### Plasmids Construct and Virus Protein Expression

PPRV genes V, H, and N were amplified from PPRV genomic cDNA and cloned into pcDNA3.1(+) (Invitrogen, V790-20). Goat NK cells were transfected with pcDNA3.1-H-HA, pcDNA3.1-N-HA, and pcDNA3.1-V-HA plasmid for 48 h and harvested and lysed; cell lysates from transfected and untransfected control cells were subjected to Western blot with antibody against HA for protein expression analysis. The empty vector pcDNA3.1 was used as a mock control.

### miRNA Mimics and Inhibitors

miR-1 mimic (miR10003322), control mimic (miR01101), miR-1 inhibitor (miR20003322), and control inhibitor (miR02101) were purchased from RiboBio (RiBoBio, Guangzhou, China).

### Transient Transfection of miRNA

Goat NK cells were grown to logarithmic phase in 96-well plates with antibiotic-free medium the day before transfection. The miRNA transfection, including miR-1 mimic, mimic control (MC), miR-1 inhibitor, and inhibitor control (NC), was performed with Lipofectamine RNAiMAX (Life Technologies, USA) on cells of 50% confluence according to the manufacturer's protocol. The final concentrations of miR-1 mimic, miR-1 inhibitor, or their negative controls (RiBoBio, Guangzhou, China) were 100 nM. The effect of transfection was examined by quantitative RT-PCR and Western blot.

### Statistical Analysis

All values are expressed as the arithmetic means of triplicates ± standard error of mean (SEM). Significance was determined by a one-way or two-way ANOVA with a Dunnett post-test, or by the Student paired *t*-test. Values of *P* < 0.05 were considered statistically significant.

## Results

### The Kinetics of Virus Replication in PPRV-Infected Goat NK Cells

**Figure 1:**
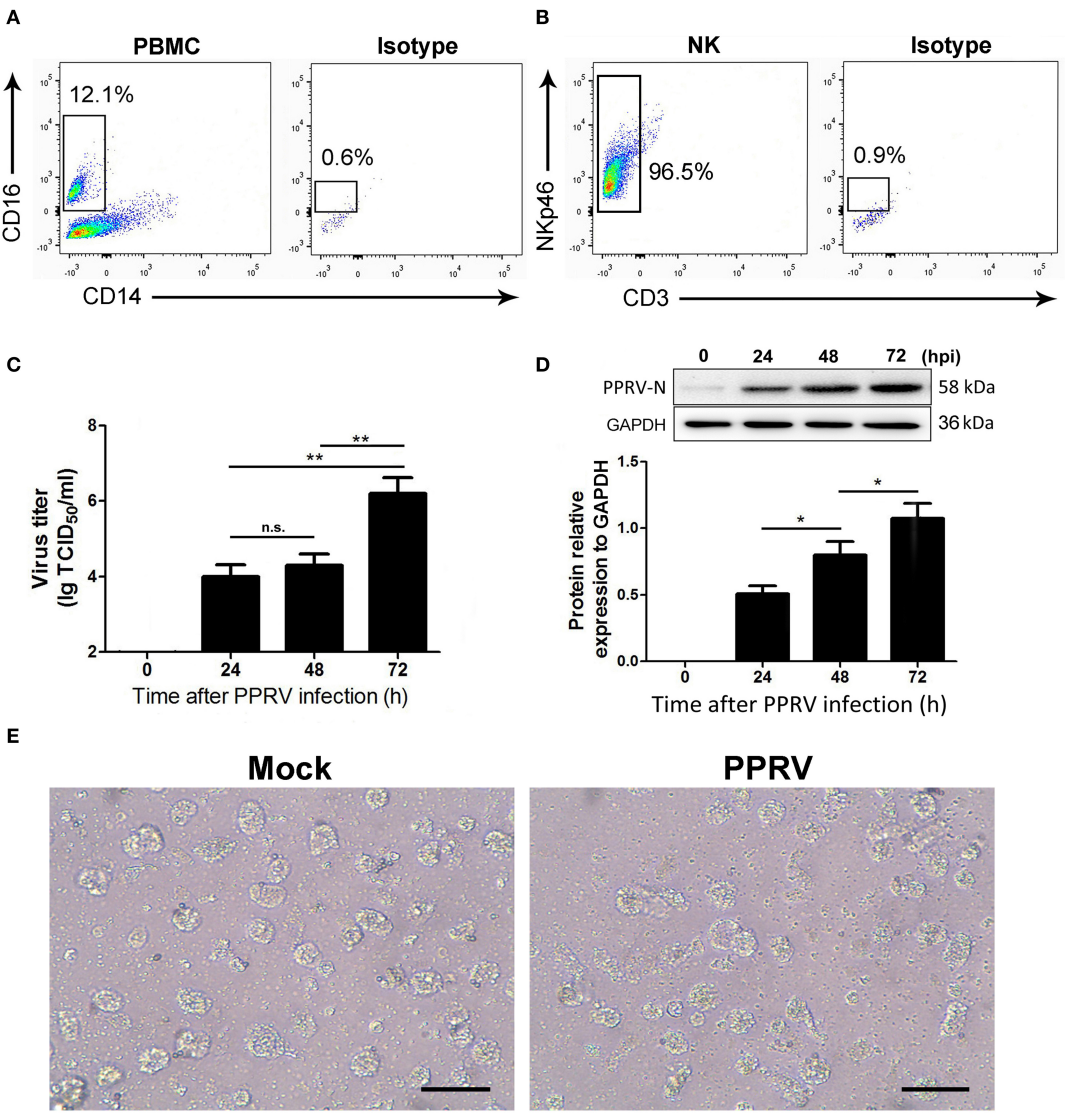
The kinetics of virus replication in PPRV-infected goat NK cells. **(A)** Representative FACS plots showing cell surface expression levels of CD16^+^CD14^−^ cells by PBMCs freshly obtained from goat. **(B)** Representative FACS plots showing freshly sorted goat CD16^+^CD14^−^ cell labeled with anti-NKp46 mAb, anti-CD3 mAb, or isotype control mAb. **(C)** Freshly isolated goat CD16^+^CD14^−^ cells were infected with PPRV at an MOI of 1 for the indicated times; the virus titers in the supernatants were measured by TCID_50_ assay. **(D)** The protein levels of PPRV-N expression in PPRV-infected CD16^+^CD14^−^ NK cells at an MOI of 1 for the indicated times were measured by Western blot. The protein levels of PPRV-N were normalized to the levels of GAPDH. **(E)** The morphology of mock- and PPRV-infected NK cells at 48 h post-infection. Results are expressed as means ± standard error of mean (SEM). *P*-values were calculated using Student's *t*-test. An asterisk indicates a comparison with the indicated control. *P < 0.05; **P < 0.01. n.s., not significant. Scale bar = 50 μm.

We identified goat NK cells as CD16^+^CD14^−^ cells as previously described (14, 15). We first analyzed the percentages of CD16^+^CD14^−^ cells in goat PBMCs and found that 10.9–12.3% of goat PBMC were CD16 positive while they do not express the monocyte marker CD14 (Figure 1A). We next verified that more than 96% of freshly sorted goat CD16^+^CD14^−^ NK cells expressed the NK cell marker NKp46 but not CD3 (Figure 1B). Thus, the isolated CD16^+^CD14^−^ cell subtype might be goat NK cells.

To determine the kinetics of PPRV replication in goat NK cells, TCID_50_ assays were performed. The time course of infected cells analysis in mock- and PPRV-infected NK cells is shown in Figure 1C. Collectively, our results revealed that an increased virus production was detected in a post-infection time-dependent manner. Importantly, a significant virus titer was detected as early as 24 hpi in PPRV-infected goat NK cells compared to mock-infected cells (Figure 1C). The results obtained from Western blot were similar with that by TCID_50_ assays (Figure 1D). We also analyzed the morphology of goat NK cells infected with PPRV and mock-infected cells (Figure 1E).

### PPRV Infection Suppresses Functional Activity of Goat NK Cells

**Figure 2:**
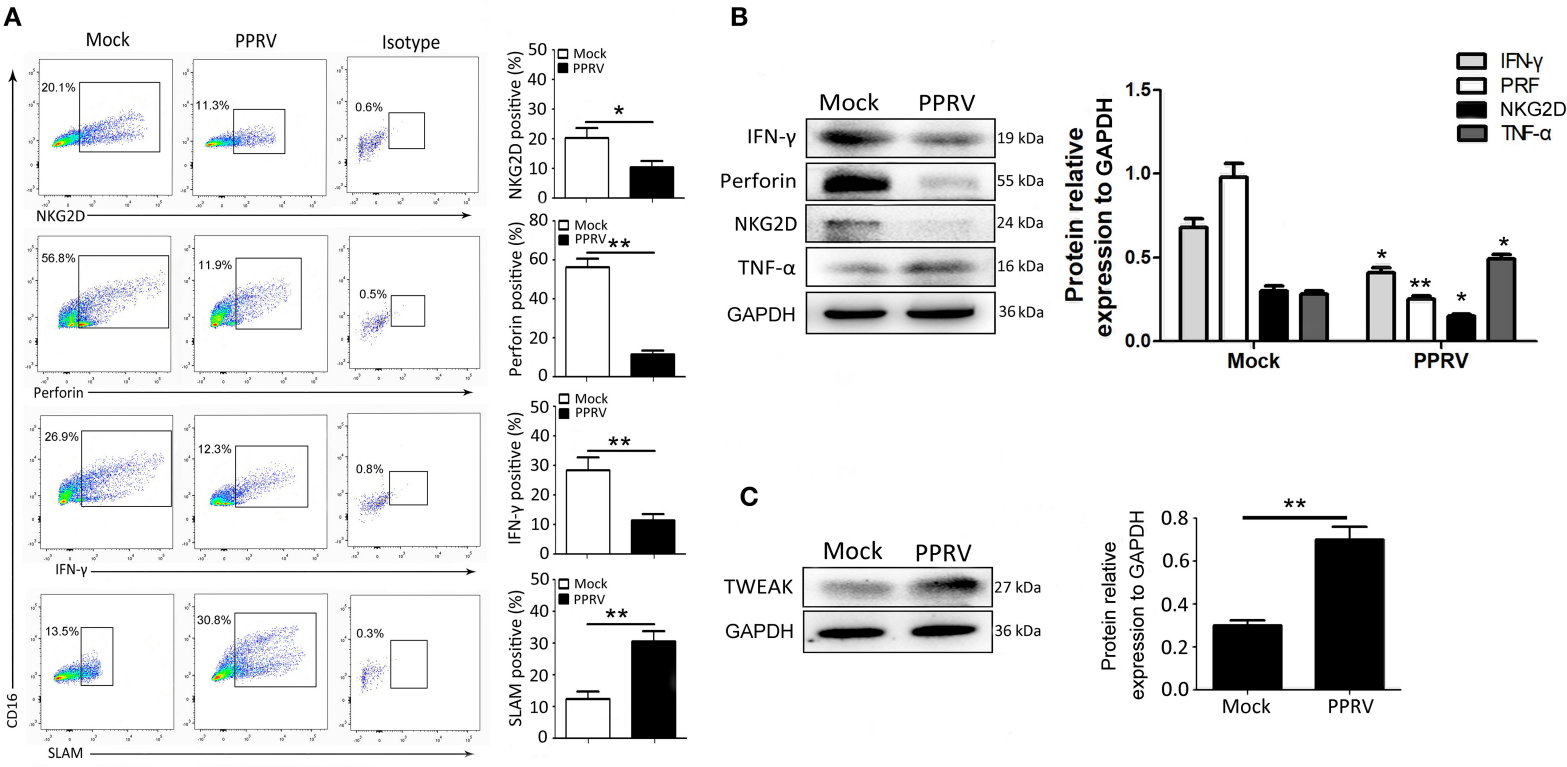
NK cell expression of cytolytic effector molecules and cytokines after PPRV infection. Goat CD16^+^CD14^−^ NK cells were infected with PPRV at an MOI of 1. On Day 1 post-infection, CD16^+^CD14^−^ NK cells were stained for cell surface expression of NKG2D, and intracellular perforin, IFN-γ, and SLAM. **(A)** Representative FACS plots for NKG2D, perforin, IFN-γ, and SLAM by CD16^+^CD14^−^-gated NK cells are shown. The numbers in the plots indicate the percentages of CD16^+^CD14^−^ NK cells that stained for NKp46^+^, perforin^+^, IFN-γ^+^, and SLAM^+^. The protein expression of NKG2D, perforin, IFN-γ, and TNF-α **(B)** as well as TWEAK **(C)** in PPRV-infected PBMCs were measured by Western blot. The protein levels of PPRV-N were normalized to the levels of GAPDH. Results are expressed as means ± standard error of mean (SEM). *P*-values were calculated using Student's *t*-test. An asterisk indicates a comparison with the indicated control. *P < 0.05; **P < 0.01.

Next, we assessed whether the immune responses of NK cells were affected by PPRV infection. As IL-12 treatment can stimulate IFN-γ production by ovine CD16^+^CD14^−^ cells (21), goat CD16^+^CD14^−^ cells were stimulated with rh IL-12 during PPRV infection. We first analyzed the expression of NKG2D, perforin, and IFN-γ in mock- and PPRV-infected goat CD16^+^CD14^−^ cells. As expected, medium or high percentages of mock-infected NK cells were positive for NKG2D, intracellular perforin, and IFN-γ (Figure 2A). However, PPRV-infected NK cells expressed sharp decreased levels of NKG2D, intracellular perforin, and IFN-γ, as compared with mock-infected cells (Figure 2A). Furthermore, we assessed the expression of SLAM on the surface of NK cells, a well-known receptor for PPRV. As shown in Figure 2A, low percentage of SLAM positive in the mock-infected group, while high levels of SLAM positive in PPRV-infected cells was detected by flow cytometry. The effect of PPRV infection on the expression of cytolytic effector molecules and cytokines by goat NK cells was also determined by Western blot assays. We found that PPRV infection suppressed the protein expression of NKG2D, perforin, and IFN-γ in NK cells compared to mock-infected cells (Figure 2B). It is well-known that TWEAK plays a prominent role in the control of the innate immune responses and TH1-based adaptive immunity transition. To examine the role of TWEAK in PPRV-infected NK cells, we performed Western blot assays to detected TWEAK expression levels in PPRV-infected goat NK cells. Interestingly, PPRV infection increased the protein level of TWEAK in the cells as compared with the mock-infected group (Figure 2C).

### TWEAK Is Critical to Goat NK Cell Activity

**Figure 3:**
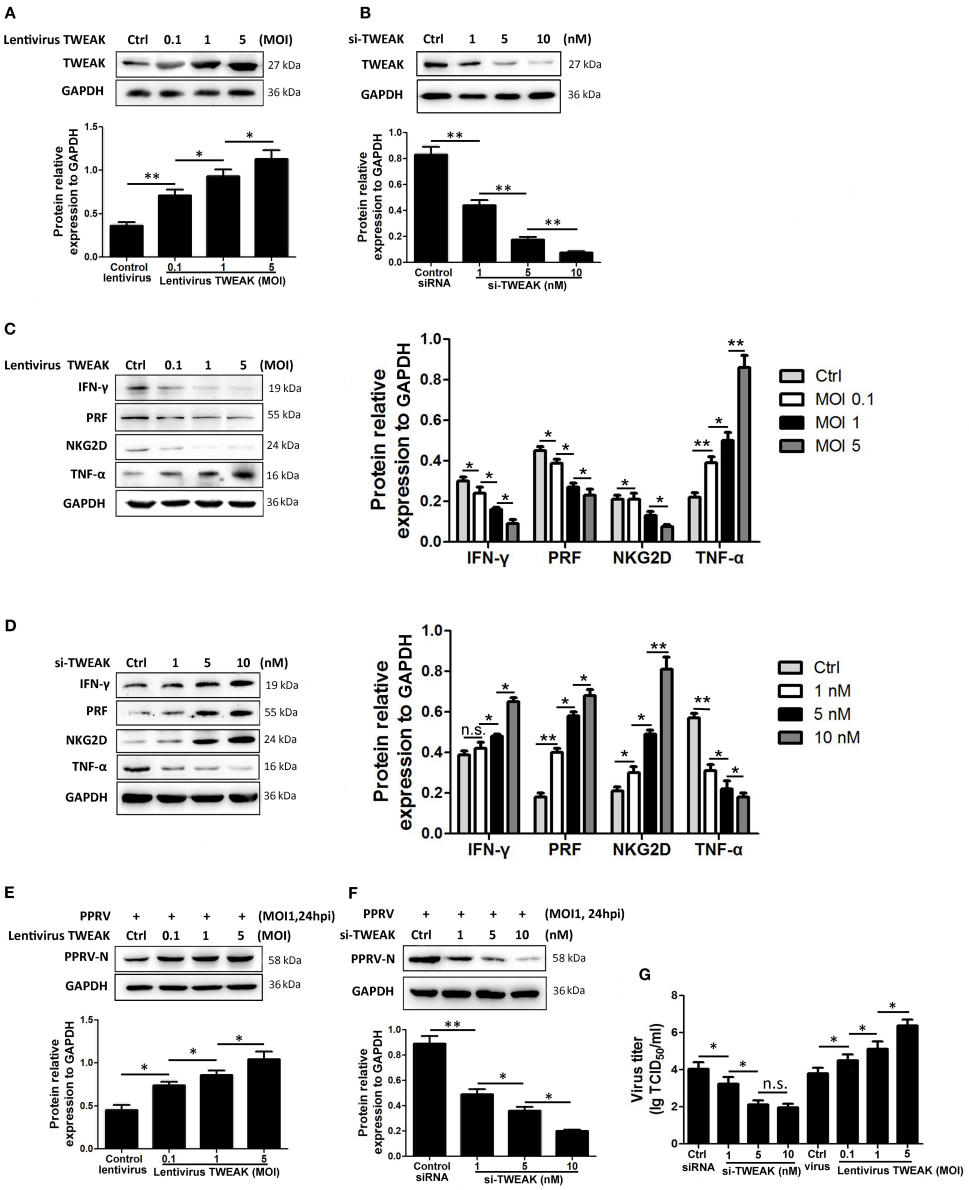
TWEAK suppresses NK cell cytolytic effector molecules and cytokines expression. **(A,B)** Goat CD16^+^CD14^−^ NK cells were transfected with lentivirus control or different MOI of lentivirus TWEAK **(A)**, or with siRNA control or different concentrations of si-TWEAK **(B)**. Forty-eight hours later, the cells were stimulated with rh IL-12, and 24 h later, the protein levels of TWEAK were determined by Western blot. **(C,D)** Goat CD16^+^CD14^−^ NK cells were transfected with different MOI of lentivirus TWEAK **(C)**, different concentrations of si-TWEAK **(D)**, or respective negative control. Forty-eight hours later, the cells were stimulated with rh IL-12, and 24 h later, the protein levels of NKG2D, perforin, IFN-γ, and TNF-α in isolated peripheral NK cells were determined by Western blot. **(E–G)** Goat CD16^+^CD14^−^ NK cells were transfected with different MOI of lentivirus TWEAK **(E)**, different concentrations of si-TWEAK **(F)**, or respective negative control. Forty-eight hours later, the cells were stimulated with rh IL-12 followed by PPRV infection at an MOI of 1, and 24 h later, the expression levels of PPRV N protein and the virus titers in the supernatants of infected cells were evaluated by Western blot **(E,F)** and TCID_50_ assay **(G)**. *P < 0.05; **P < 0.01. n.s., not significant.

To investigate the role of TWEAK in the regulation of NK cell activity, freshly isolated goat CD16^+^CD14^−^ NK cells were transfected with siRNA targeting TWEAK gene (si-TWEAK), TWEAK overexpression lentivirus, or respective negative control. Forty-eight hours later, the cells were stimulated with rh IL-12 as previously described (21), and 24 h later, the protein levels of TWEAK as well as NKG2D, perforin, IFN-γ, and TNF-α in NK cells were determined by Western blot. As expected, the cells infected with lentivirus TWEAK resulted in a significant dose-dependent upregulation of TWEAK as compared to negative control (Figure 3A). However, knockdown of TWEAK with si-TWEAK resulted in the downregulation of TWEAK expression (Figure 3B). Importantly, TWEAK upregulation suppressed the expression of NKG2D, perforin, and IFN-γ in primary goat NK cells, while it stimulated TNF-α expression in a dose-dependent manner (Figure 3C). However, an opposite effect was observed in cells transfected with siRNAs targeting TWEAK (Figure 3D). Additionally, to investigate the role of TWEAK in the regulation of PPRV replication and virus progeny, the above transfected cells were stimulated with IL-12 followed by PPRV infection at an MOI of 1 for 24 h. Then, PPRV N protein in infected NK cells and the virus titers in the supernatants were evaluated by Western blot and TCID_50_, respectively. The results showed that upregulation of TWEAK facilitated the replication of PPRV in the goat NK cells (Figures 3E,G), while downregulation of TWEAK inhibited the replication of virus (Figures 3F,G).

### TWEAK Expression Is Negatively Associated With miR-1 Expression Levels During PPRV Infection

**Figure 4:**
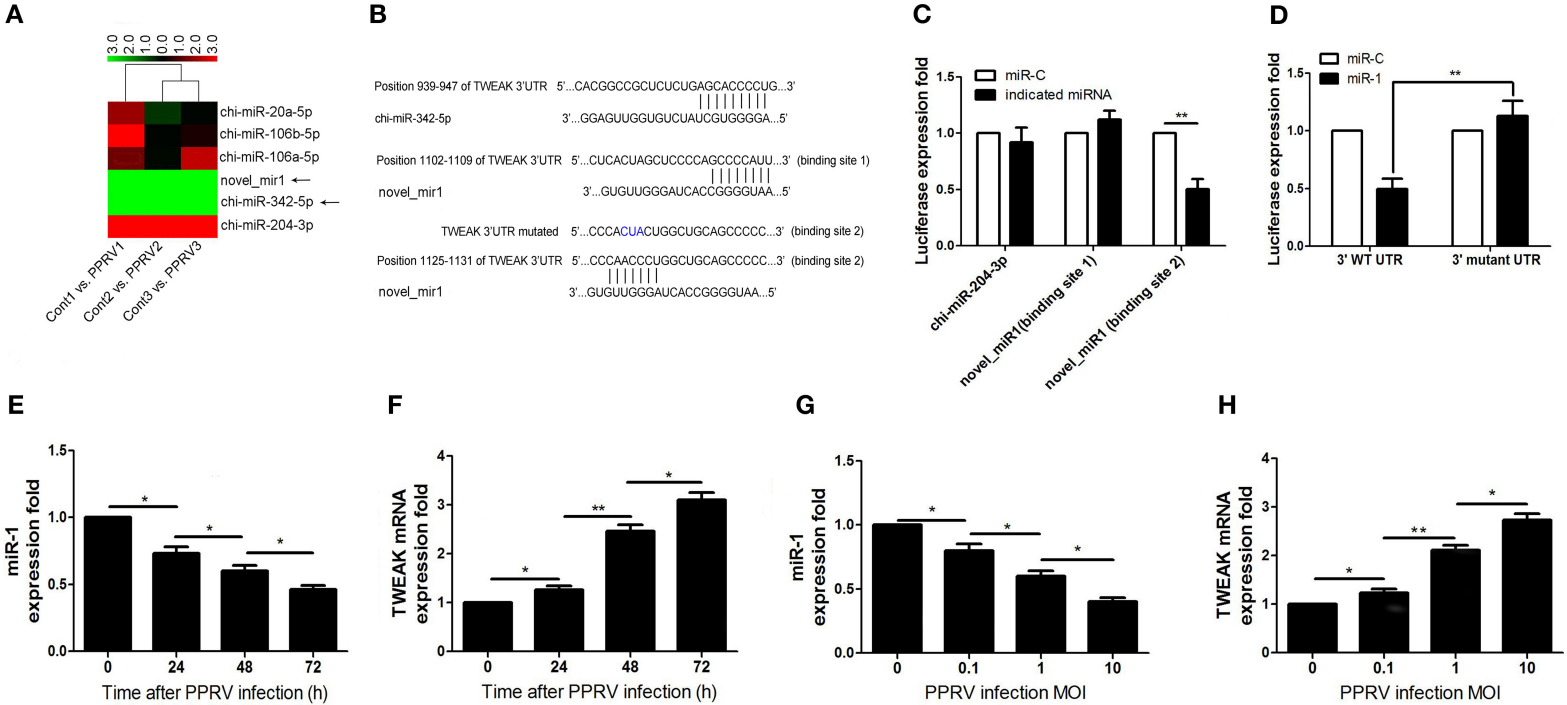
miR-1 targets TWEAK in PPRV-infected goat PBMCs. **(A)** Heatmap of miRNAs predicted targets TWEAK in PPRV-infected goat PBMCs vs. mock-infected cells. **(B)** Predicted interaction between screened miRNAs and the putative binding sites in the TWEAK 3′UTRs. **(C)** Dual-luciferase assay of HEK293T cells transfected with luciferase constructs containing the putative chi-miR-204-3p, or the two putative miR-1-binding sites (binding site 1 or binding site 2) or a synthetic control miRNA with a scrambled sequence (miR-C). **(D)** Dual-luciferase assay of HEK293T cells transfected with luciferase constructs containing the wild-type 3′UTRs (3′WT UTRs) or mutated 3′UTRs (with deletion of the miR-1-responsive element) from TWEAK, plus miRNA. **(E–H)** Goat CD16^+^CD14^−^ NK cells were infected with PPRV at an MOI of 1 for the indicated times **(E,F)**, or at different MOIs for 24 h **(G,H)**, and the expression of miR-1 **(E,G)** and TWEAK mRNA expression **(F,H)** were measured by qRT-PCR. *P < 0.05; **P < 0.01.

Although the changing expression of TWEAK evidently contributed to modulate functional activity of peripheral NK cells during PPRV infection, the underlying mechanisms that controlled TWEAK expression largely remain unknown. Our previous study has identified 316 significantly differentially expressed miRNAs, including 103 known and 213 novel miRNAs, from PPRV-infected goat PBMCs as compared with mock control (27). Here, we identified differentially expressed miRNAs that were predicated by TargetScan to target the *TWEAK* gene and their fold change. Among these miRNAs, we found six miRNAs target to *TWEAK* gene (Table 1 and Figure 4A). We focused on chi-miR-342-5p and novel_miR1 because both the expression in infected cells decreased significantly relative to mock-infected cells (Table 1 and Figure 4A). Then, the putative binding sites in the *TWEAK* 3′UTRs were identified according to the TargetScan algorithm (Figure 4B). To investigate which putative binding sites were functional, the fragment of putative miRNA binding sites contained in *TWEAK* 3′UTRs mRNA (XM_018064586.1) was cloned. Then, the constructed vectors and mature chi-miR-342-5p or novel_miR1 transfected into HEK293 cells, or a control miRNA with a scrambled sequence were transfected into HEK293 cells. The results showed that miR-1 significantly inhibited the relative luciferase activity of the binding site 2 contained vector in comparison with control. However, no inhibitory effect was detected on the activity of other binding sites (Figure 4C). Furthermore, the inhibitory effect was abrogated in cells transfected with responsive elements deleted miR-1 and *TWEAK* 3′UTRs (Figure 4D). To confirm this result, the kinetics of miR-1 and TWEAK mRNA expression in goat NK cells infected with PPRV were analyzed by qRT-PCR. Collectively, an inverse correlation between the expression of miR-1 and TWEAK mRNA following PPRV infection was confirmed either in a post-infection time-dependent (Figures 4E,F) or in a virus dose-dependent manner (Figures 4G,H).

**Table 1:** Differentially expressed cellular microRNAs predicted targets TWEAK gene in PPRV- vs. mock-infected goat PBMC.

**miRNA name**	**Sequence (5^′^-3^′^)**	**Up-/downregulation**	**Expression fold change**
chi-miR-20a-5p	uaaagugcuuauagugcagguag	UP	2.886
chi-miR-106b-5p	uaaagugcugacagugcagau	UP	3.584
chi-miR-106a-5p	aaaagugcuuacagugcagguagc	UP	3.962
novel_mir1	aauggggccacuaggguugug	DOWN	−3.918
chi-miR-342-5p	aggggugcuaucugugguugagg	DOWN	−3.362
chi-miR-204-3p	gcugggaaggcaaagggac	UP	4.583

### miR-1 Regulates TWEAK Expression and Virus Replication in PPRV-Infected Goat NK Cells

**Figure 5:**
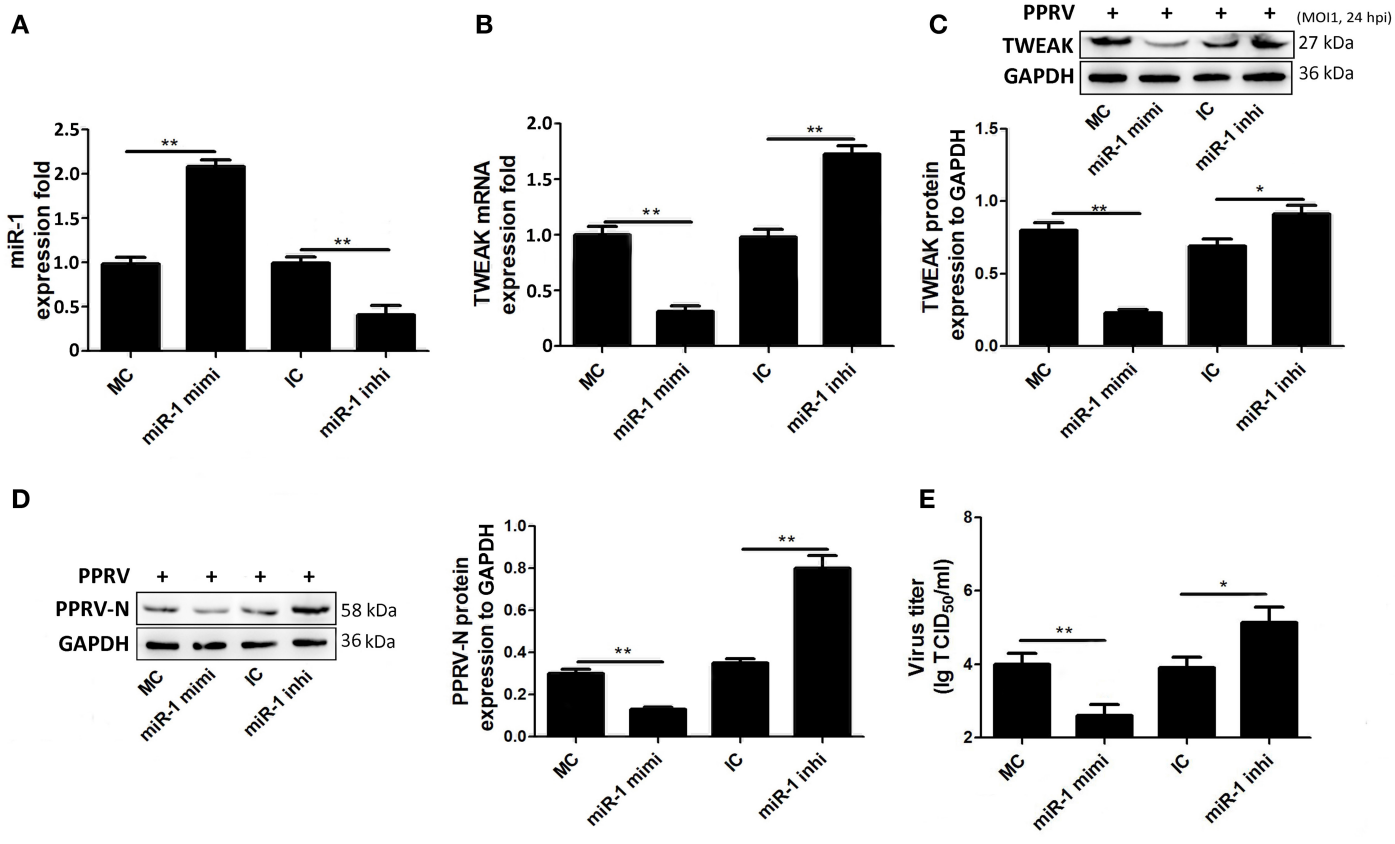
miR-1 suppresses TWEAK expression and virus replication during PPRV infection. **(A–C)** Goat CD16^+^CD14^−^ NK cells were transfected with control miRNA (MC) or miR-1 mimics, or control inhibitor (IC) or miR-1 inhibitor, as indicated at a final concentration of 50 nM. Forty-eight hours later, the cells were infected with PPRV at an MOI of 1, and 24 h later, miR-1 **(A)** and TWEAK mRNA **(B)** expression was measured by qRT-PCR and the protein levels of TWEAK were determined by Western blot **(C)**. **(D,E)** Goat CD16^+^CD14^−^ NK cells were transfected with miR-1 mimics, miR-1 inhibitor, or respective negative control. Forty-eight hours later, the cells were infected with PPRV at an MOI of 1, and 24 h later, the expression levels of PPRV N protein and the virus titers in the supernatants of infected cells were evaluated by Western blot **(D)** and TCID_50_ assay **(E)**. The levels of miR-1 were normalized to the levels of 5S. The levels of TWEAK and PPRV-N were normalized to the levels of GAPDH. Results are expressed as means ± standard error of mean (SEM). *P*-values were calculated using Student's *t*-test. An asterisk indicates a comparison with the indicated control. *P < 0.05; **P < 0.01.

To further test miR-1, goat NK cells were transfected with miR-1 mimic or miR-1 inhibitor, and 48 h later, the cells were infected with PPRV at a MOI of 1. After 24 h infection of PPRV, the expression of miR-1 and TWEAK was examined by qRT-PCR. As expected, the expression of miR-1 in cells transfected with miR-1 mimic significantly increased compared to mimic negative control (MC), while transfection of miR-1 inhibitor decreased miR-1 expression compared to inhibitor negative control (IC) (Figure 5A). Furthermore, we observed that pretreatment with miR-1 mimic led to a reduced TWEAK mRNA and protein expression levels as compared with that of MC (Figures 5B,C), while a significant increased TWEAK expression was detected in miR-1 inhibitor transfected cells compared to IC (Figures 5B,C). In order to explore the role of miR-1 in the replication of PPRV, Western blot and TCID_50_ assays were performed, and the results showed that miR-1 mimic downregulated the levels of PPRV N protein (Figure 5D) and the virus titers (Figure 5E), while miR-1 inhibitor enhanced the levels of PPRV N protein (Figure 5D) and the virus titers (Figure 5E). Altogether, these results clearly showed that cellular miR-1 have negative effects on TWEAK expression and viral replication in PPRV-infected NK cells.

### NK Cell Function Is Associated With miR-1 Expression Levels During PPRV Infection

**Figure 6:**
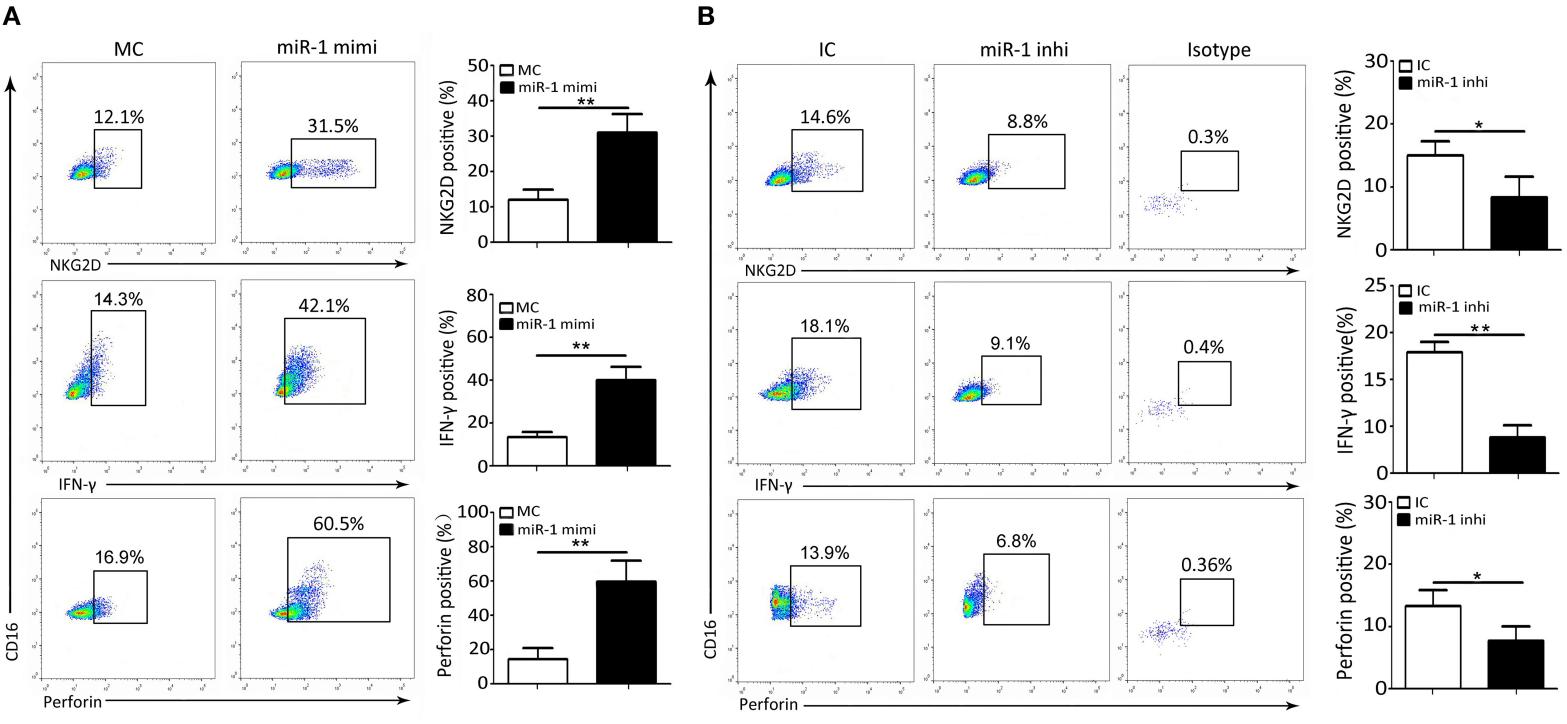
miR-1 suppresses peripheral NK cell cytolytic effector molecules and cytokine expression during PPRV infection. **(A,B)** Goat CD16^+^CD14^−^ NK cells were transfected with control miRNA (MC) or miR-1 mimics **(A)**, or control inhibitor (IC) or miR-1 inhibitor **(B)**, as indicated at a final concentration of 50 nM. Forty-eight hours later, the cells were infected with PPRV at an MOI of 1, and 24 h later, cells were stained for cell surface expression of NKG2D, IFN-γ, and intracellular perforin. Representative FACS plots for NKG2D, IFN-γ, and perforin by CD16^+^CD14^−^ NK cells are shown. The numbers in the plots indicate the percentages of CD16^+^CD14^−^ NK cells that stained for NKG2D^+^, IFN-γ^+^, and perforin^+^. *P < 0.05; **P < 0.01.

Next, we further ascertained the role of miR-1 in the expression of cytolytic effector molecules and cytokines expression by NK cells infected with PPRV. To this end, freshly isolated goat NK cells were pre-transfected with miR-1 mimic, miR-1 inhibitor, or respective control for 48 h followed by PPRV infection for 24 h. Our results showed that, compared with MC, pretreatment with miR-1 mimic increased percentage of NKG2D, IFN-γ, and perforin-positive NK cells (Figure 6A). However, the percentage of NKG2D, IFN-γ, and perforin-positive NK cells significantly decreased in NK cells transfected with miR-1 inhibitor as compared with IC (Figure 6B). These results were similar to the results obtained from the overexpression of TWEAK by lentivurus TWEAK infection or knockdown by siRNA targeting TWEAK (Figures 3C,D).

### Replication of PPRV Is Required for Inhibition of miR-1 Expression

**Figure 7:**
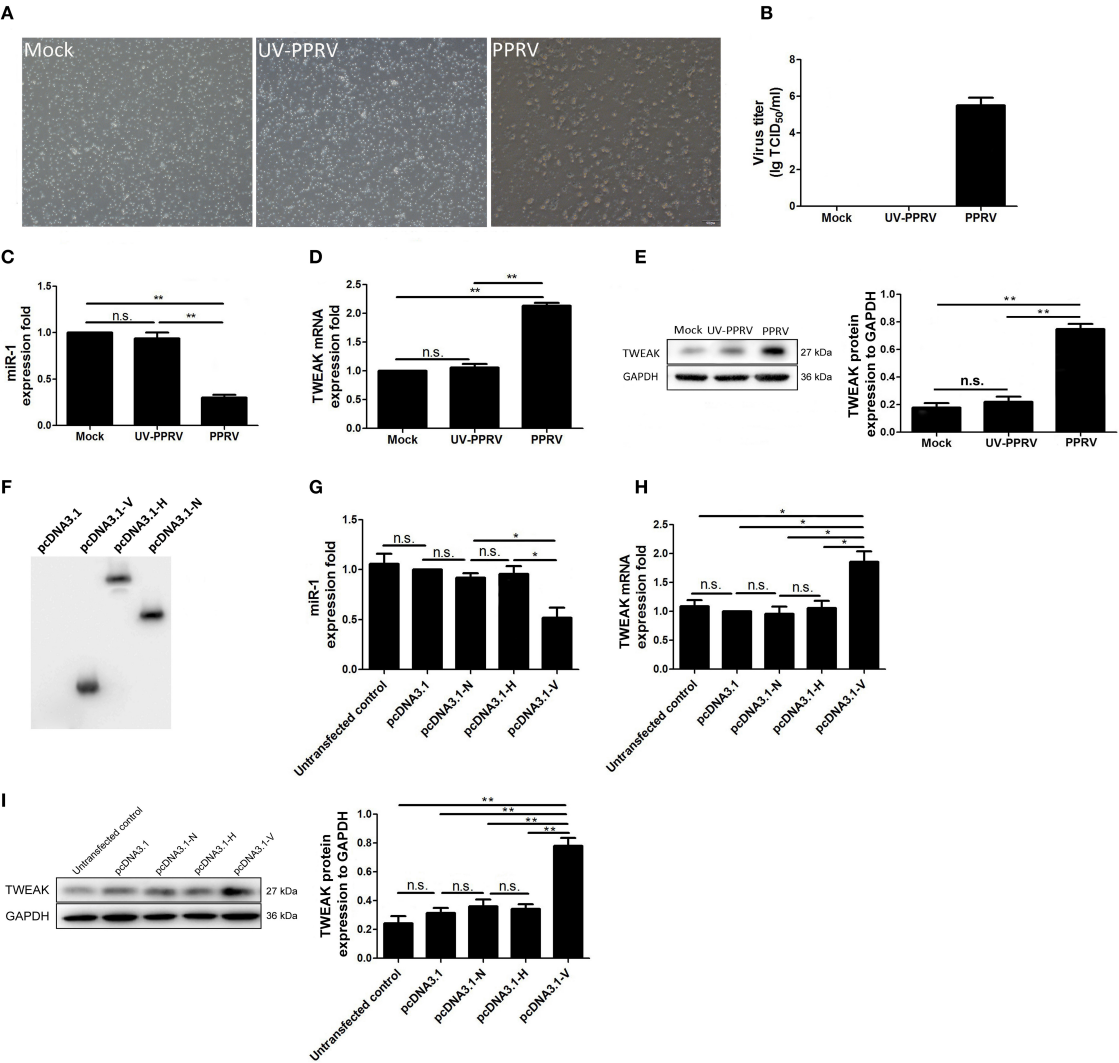
Replication of PPRV is required for inhibition of miR-1 expression. **(A,B)** Goat PBMCs were mock-infected or infected with PPRV (MOI = 1) or UV-inactivated PPRV (MOI = 1) for 72 hpi. At the end of the infection, cytopathic effect (CPE) **(A)** was detected and virus titers were measured by using the TCID_50_ method **(B)**. **(C–E)** Goat CD16^+^CD14^−^ NK cells were mock infected, infected with PPRV or with UV-inactivated (UV-PPRV) at an MOI of 1 for 24 h, and the levels of miR-1 **(C)** and TWEAK mRNA **(D)** were detected by qRT-PCR, while TWEAK protein expression was measured by Western blot **(E)**. Histograms of the right panel showed densitometric analysis of indicated protein normalized to GAPDH to correct for protein loading. **(F)** Goat CD16^+^CD14^−^ NK cells were transfected with pcDNA3.1-HA, pcDNA3.1-H-HA, pcDNA3.1-N-HA, and pCDNA3.1-V-HA plasmid for 48 h, and cell lysates from transfected cells were subjected to Western blot with antibody against HA. **(G)** Goat CD16^+^CD14^−^ NK cells were transfected with pcDNA3.1-HA, pCDNA3.1-H-HA, pCDNA3.1-N-HA, and pCDNA3.1-V-HA plasmid for 48 h, and miR-1 expression in transfected and untransfected control cells was subjected to qRT-PCR analysis. **(H,I)** Goat CD16^+^CD14^−^ NK cells were transfected with pcDNA3.1-HA, pCDNA3.1-H-HA, pCDNA3.1-N-HA, and pCDNA3.1-V-HA plasmid for 48 h, and the expression of TWEAK in transfected and untransfected control cells was subjected to qRT-PCR **(H)** and Western blot analysis **(I)**. The levels of miR-1 were normalized to the level of 5S. GAPDH was used as a loading control in Western blot analysis. Results are expressed as means ± standard error of mean (SEM). *P*-values were calculated using Student's *t*-test. An asterisk indicates a comparison with the indicated control. *P < 0.05; **P < 0.01. n.s., not significant.

To explore whether viral replication plays a role in miR-1-mediated TWEAK expression in PPRV-infected goat NK cells, live PPRV was inactivated by ultraviolet (UV) irradiation and its capability for modulation of miR-1 and TWEAK expression was determined. Our data showed that active PPRV infection caused a clear cytopathic effect (CPE) (Figure 7A) and virus titers (Figure 7B) in PBMCs but not UV-inactivated PPRV infected cells at 72 hpi, which indicate that UV-inactivated PPRV lost its ability to infect cells. Furthermore, the expression of miR-1 was downregulated by 2.9-fold in response to PPRV infection compared to mock infection, while no significant difference in miR-1 expression was observed in UV-PPRV-infected goat NK cells (Figure 7C). We further investigated the expression of TWEAK by qRT-PCR and Western blot assay. As expected, both the mRNA (Figure 7D) and protein expression (Figure 7E) of TWEAK gene have inverse correlation with miR-1 expression (Figure 7C) in mock-, PPRV-, and UV-PPRV-infected cells.

PPRV non-structural protein V has previously been demonstrated to block type I and type II interferon signaling pathways (28, 31, 32). To determine whether V protein could cause miR-1-mediated upregulation of TWEAK, goat NK cells were transfected with empty vector pcDNA3.1, pcDNA3.1-H-HA, pcDNA3.1-N-HA, and pcDNA3.1-V-HA plasmid for 48 h and harvested and lysed; cell lysates were subjected to Western blot with antibody against HA for the analysis of the expression of PPRV N, H, and V in goat NK cells (Figure 7F). The presence of the HA tag at the C terminus of H protein did not affect the protein's function (data not shown). Our data demonstrated that V protein of PPRV reduced miR-1 expression (Figure 7G) and upregulated TWEAK expression in goat NK cells (Figures 7H,I). Together, these experiments indicated that PPRV V protein alone was sufficient to cause the increased TWEAK expression through downregulation miR-1 expression in goat NK cells.

### Regulation of NK Cell Function by miR-1 Is Mediated by TWEAK Signaling

**Figure 8:**
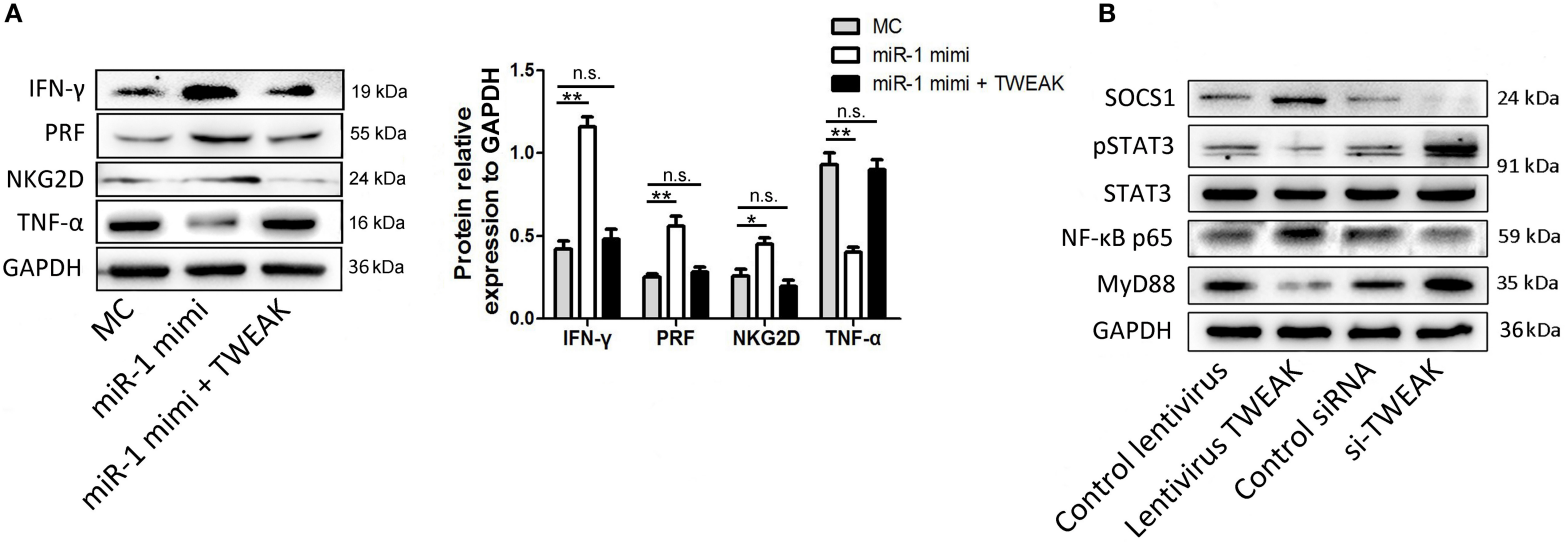
Regulation of NK cell function by miR-1 is mediated by TWEAK signaling. **(A)** Goat CD16^+^CD14^−^ NK cells were transfected with control miRNA (MC) or miR-1 mimics, or miR-1 mimics and incubated with recombinant TWEAK at the final concentration of 50 nM. Forty-eight hours later, the cells were infected with PPRV at an MOI of 1, and 24 h later, the protein expression of NKG2D, perforin, IFN-γ, and TNF-α was measured by Western blot. Histograms of the right panel showed densitometric analysis of indicated protein normalized to GAPDH to correct for protein loading. **(B)** Goat CD16^+^CD14^−^ NK cells were transfected with lentivirus TWEAK, si-TWEAK, or respective negative control. Forty-eight hours later, the cells were infected with PPRV at an MOI of 1, and 24 h later, the protein levels of SOCS-1, MyD88, STAT3, pSTAT3, and NF-κB p65 in NK cells were determined by Western blot and normalized to the levels of GAPDH. Results are expressed as means ± standard error of mean (SEM). *P*-values were calculated using Student's *t*-test. An asterisk indicates a comparison with the indicated control. *P < 0.05; **P < 0.01. n.s., not significant.

To test the hypothesis that regulation of NK cell function by miR-1 is TWEAK mediated, we subsequently added recombinant TWEAK to the miR-1 mimic pre-transfected NK cells following PPRV infection. As indicated by Western blot results, supplement with TWEAK reversed the increased function activity in miR-1 mimic transfected NK cells (Figure 8A). Thus, we conclude that miR-1 acts through TWEAK to regulate NK cell function during PPRV infection.

TWEAK signaling was reported previously to suppress the production of IFN-γ by NK cells through suppressor of cytokine signaling (SOCS)-1 induction. To test the underlying mechanisms of inhibited IFN-γ production by TWEAK in goat NK cells, we primarily examined the effect of TWEAK on SOCS-1 expression in goat NK cells. Our data showed that TWEAK overexpression by infected lentivirus TWEAK increased the levels of SOCS-1, while TWEAK knockdown by siRNA targeting TWEAK decreased the levels of this gene, as compared to respective control (Figure 8B). Since STAT3 activation is positively correlated with basal NKG2D expression and cytolytic activity, we also determined whether TWEAK can induce STAT3-mediated NKG2D expression and cytolytic activity of NK cells. Our results showed that TWEAK overexpression decreased basal levels of phosphor-STAT3 as compared to control, while increased phosphor-STAT3 levels were observed in TWEAK knockdown cells (Figure 8B). We also determined whether TWEAK's modulation of the immune functions of NK cells may involved induced association of NF-κB p65 and MyD88, since PPRV-induced TWEAK expression increased the expression of TNF-α of NK cells. Our data showed that overexpression of TWEAK enhances NF-κB p65 expression and attenuated MyD88 expression in NK cells, while opposite effects were observed in TWEAK knockdown cells, as compared to respective control (Figure 8B).

**Figure 9:**
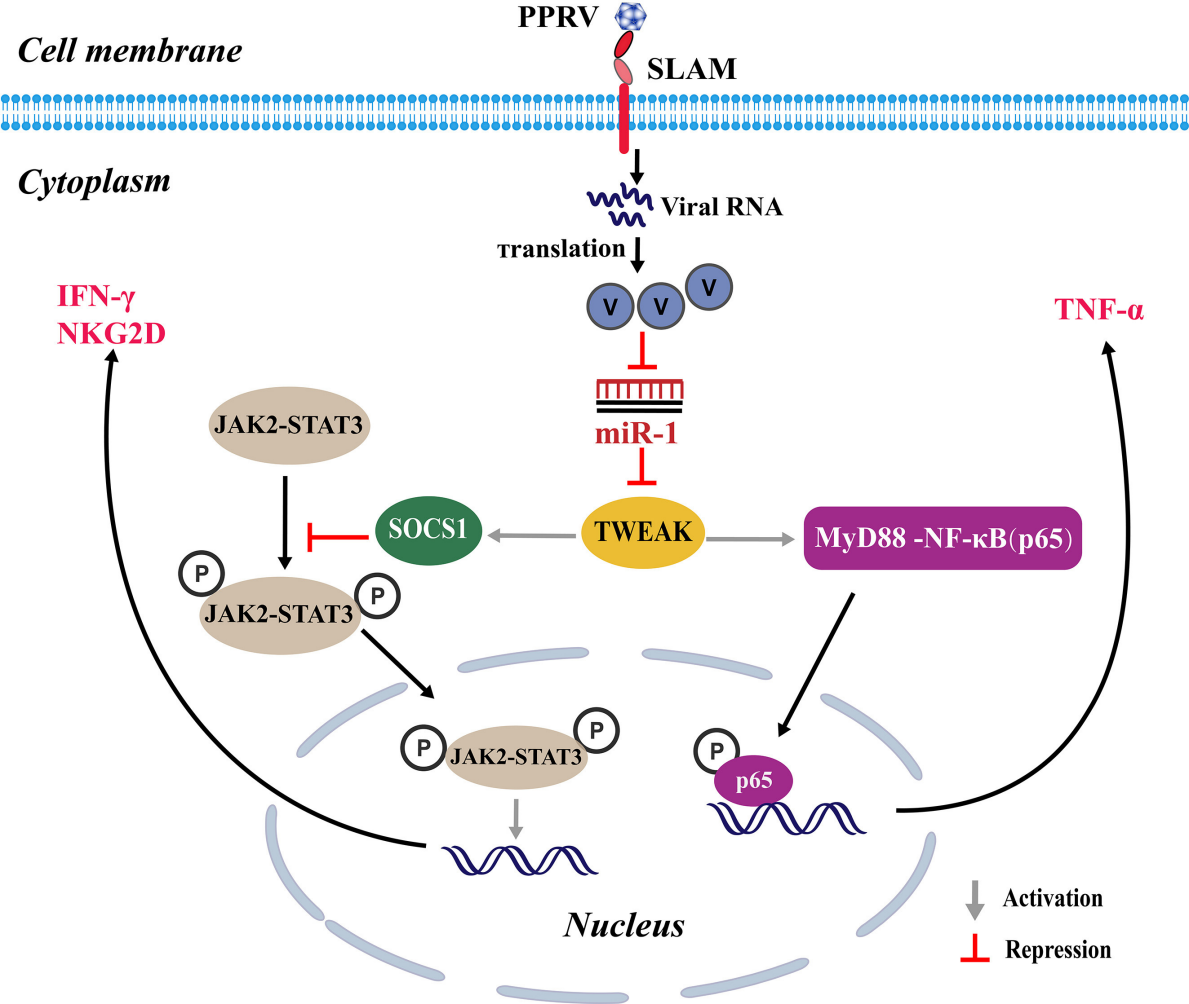
Proposed model for the inhibition of miR-1 expression upon PPRV infection and the role of miR-1 in the regulation of the NK cell immune responses. During PPRV infection, TWEAK expression in goat NK cells is stimulated through suppressing miR-1, which in turn suppresses NK cell cytotoxicity and cytokine expression in a SOCS1 and NF-κB-dependent manner. The non-structural V protein of PPRV plays an important role in miR-1-mediated TWEAK upregulation.

Collectively, our findings revealed that PPRV infection significantly induces TWEAK expression in goat NK cells through suppressing miR-1, which in turn suppresses NK cell cytotoxicity and cytokine expression in a SOCS1 and NF-κB-dependent manner. Moreover, we revealed that the non-structural V protein of PPRV plays an important role in miR-1-mediated TWEAK upregulation (Figure 9).

## Discussion

PPRV infection causes serious consequences for the host, including suppression of the immune system, especially innate immunity. In this study we analyzed the mechanisms involved in immunosuppression during PPRV infection in goat peripheral NK cells for the central role TWEAK. Here, we reveal that multiple immune responses of goat NK cells were compromised during PPRV infection, including cytolytic effector molecule expression and cytokine production. Subsequently, we demonstrated that increased TWEAK expression in PPRV-infected NK cells suppressed cytotoxicity as well as cytokine expression, and TWEAK acted as a negative regulator for NK cells during PPRV infection. Furthermore, we found that cellular miR-1 functions as a critical regulator of goat NK cell cytotoxic activity and cytokine production by directly targeting TWEAK gene. Importantly, there was a positive correlation between miR-1 expression and viral loads in PPRV-infected NK cells. Moreover, we reveal that regulation of NK cell immune responses by TWEAK is mediated by NF-κB p65, SOCS1, and STAT3.

NK cells represent the first line of the host defense against invading pathogens. However, it remains largely unknown how NK cells respond and are regulated at the earliest time points after an acute PPRV infection in goats. Here, we clearly show that peripheral NK cells mounted a compromised response to acute experimental PPRV infection. Our data revealed a reduced cytolysis activity and cytokine production of NK cells in response to PPRV early infection, indicating that the NK cells undergo an inhibitory function following PPRV infection. Previous studies have demonstrated that vaccine PPRV induced a transient lymphopenia in goat (7). Here, we clearly show that immune responses of peripheral NK cells are severely impaired after acute attenuated PPRV infection.

It has been demonstrated that the specific cytokine environment affects NK cell function exposed (22). PPRV was shown to alter a large number of innate immune response-related gene expression, with a peak in gene expression alteration at early infection (40). It remains largely unknown as yet whether generation of cytokine environment during PPRV infection affects NK cell function exposed. TWEAK protein is expressed broadly by peripheral blood-derived innate immune cells, including polymorphonuclear leukocytes, macrophages, DCs, and NK cells, as well as in B- and T-cell subsets. Previous studies have indicated that sequence similarity at the amino acid level between bovine and human or mouse TWEAK is 95 and 92%, respectively, with a sequence identity of 91 and 88%, respectively, suggesting that the TWEAK gene has been highly conserved during evolution (41). In the current study, we revealed that PPRV infection induced TWEAK expression in goat NK cells, which was accompanied by a suppression of functional activity, as well as the increase in viral load. Further analysis showed that functional activity of NK cells was negatively associated with the expression levels TWEAK. The importance of TWEAK is emphasized by the fact that some viruses, including human HCV and KSHV, can increase the constitutive levels of TWEAK to facilitate their infections (42, 43). Furthermore, studies in humans and mice have demonstrated that TWEAK can prevent local cytotoxicity and counterbalance the cytotoxic function of NK cells during the early phase of infection (44–46). Interferon-γ is primarily produced by NK cells and play a role in shaping the T-helper 1 (Th1) immune responses. Previous work from our laboratory showed that TWEAK decreases the expression of IFN-γ and the cytotoxicity of goat uterine NK cells, a major lymphocyte population in the endometrium of goat (47, 48). Here, our data also support a negative role of TWEAK in regulating peripheral NK cell IFN-γ production. NKG2D-mediated immune response plays a critical role for NK cells to infections and tumors (13). Although the regulation of NKG2D ligand has been fully investigated (49), little is known about the mechanisms of NKG2D receptor regulation. Our data showed that NKG2D expression on NK cells is downregulated in response to stimulated TWEAK expression. Collectively, we clearly showed that PPRV infection stimulated TWEAK expression in goat NK cells and was involved in the dysfunction of peripheral NK cells. Thus, although further investigation of this aspect is warranted, regulation of TWEAK expression may be an important mechanism for regulating NK cell function in ruminants.

MicroRNAs have been reported to regulate the innate and acquired immune responses, as well as the differentiation and development of immune cells. A growing number of reports suggest that virus infection, including PPRV, can alter cellular miRNA expression profile and thereby regulate host or viral RNA targets (50–52). Recently, our group has identified 103 known and 213 novel miRNAs from PPRV-infected goat PBMCs by using small RNA deep sequencing (27). Here, we demonstrated that miR-1 can downregulate TWEAK expression through directly targeting its mRNA, and more importantly, there was an inverse correlation between the expression of miR-1 and TWEAK during PPRV infection in a viral dose- or post-infection time-dependent manner. In addition, the experiment with miR-1 mimics and miR-1 inhibitor further demonstrated that miR-1 suppressed the production of TWEAK during PPRV infection as well as the viral loads. However, whether miRNAs other than miR-1 participate in PPRV-mediated evasion of NK cell-mediated antiviral responses remains elusive. Moreover, the experiment with miR-1 mimics and miR-1 inhibitor further demonstrated that miR-1 expression has positive effects on the percentage of NKG2D, IFN-γ, and perforin-positive cells. Combined with the data obtained from the overexpression of TWEAK by lentivurus TWEAK infection or knockdown by siRNA targeting TWEAK, these results clearly indicated that PPRV infection downregulates the expression of miR-1 and hijacks the host miR-1 to modulate TWEAK expression and promote virus production. Furthermore, transfection of miR-1 mimic induced changed expression of cytotoxic activity and cytokines of peripheral NK cells could be rescued by treatment with recombinant TWEAK, further confirming that PPRV-decreased miR-1 is responsible for the increased TWEAK.

Furthermore, to elucidate whether the replication of PPRV is required for the inhibition of miR-1 levels, we further infected goat NK cells with either replication-competent or UV-inactivated PPRV and then measured their effects on the miR-1 and TWEAK expression. Although an inverse correlation between the expression of miR-1 and TWEAK was observed in the replication-competent PPRV group, there was no significant difference between UV-inactivated PPRV and mock group either in miR-1 or TWEAK expression. These results suggest that active PPRV replication is required for miR-1-mediated TWEAK expression. There is extensive evidence that PPRV V protein is involved in controlling both type I and type II interferon (IFN) action (28, 31, 32). Here, our data showed that transfection of NK cells with pcDNA3.1-V-HA plasmid alone can upregulate the expression of TWEAK in cells. In this process, it seems that miR-1 involved for decreased expression levels of miR-1 was detected in PPRV V protein alone treated cells. This suggests that the non-structural V protein of PPRV plays an important role in miR-1-mediated TWEAK upregulation during PPRV infection.

STAT3 is considered as a key regulator of innate and adaptive immune responses. Previous studies have demonstrated that defects in STAT3 signaling result in deficient NKG2D responses to cytokine in human NK cells (14). Some viruses of *Paramyxoviriuses*, including PPRV, interfere IFN actions by disturbing STAT protein distribution (28, 31, 32). Here, overexpression of TWEAK attenuated PPRV-induced STAT3 phosphorylation in goat NK cells and augmented SOCS1, a negative-feedback regulator of STAT activation. Thus, TWEAK may suppress the production of IFN-γ and NKG2D expression by inhibiting STAT3 activation through upregulating SOCS1. Moreover, PPRV-induced TWEAK expression attenuated the production of IFN-γ and cytotoxicity but increased the expression of TNF-α of NK cells. Thus, a second mechanism contributing to TWEAK's modulation of the immune functions of NK cells may involve the induced association of NF-κB p65 and MyD88. This is further supported by observations in this study that overexpression of TWEAK enhances NF-κB p65 expression and attenuated MyD88 expression in NK cells, while opposite effects were observed in TWEAK knockdown cells. These findings suggest that the expression of TWEAK in response to PPRV infection suppresses the immune functions of peripheral NK cells, and the underlying mechanisms may involve repression of STAT3 activation and promotion of MyD88-NF-κB p65 signaling in NK cells. Further studies needed to address whether other STAT-family members also regulate NKG2D and IFN-γ production, either independent of or as heterodimers with STAT3.

In summary, the findings presented here suggest a previously unrecognized role for TWEAK in the regulation of goat peripheral NK cell cytotoxicity and cytokine expression during acute PPRV infection. Targeting TWEAK has the potential to not only direct suppress virus replication but also alter the host cell immunologic environment in favor of immunotherapy. With the emergence of TWEAK inhibitors, both indirect and direct, TWEAK can be used as a promising target for antiviral therapy.

## Data Availability Statement

All datasets generated for this study are included in the article/supplementary material.

## Ethics Statement

The animal experiments were carried out in strict accordance with guidelines established by the Ethics of Animal Experiments of Northwest A&F University, Yangling, China. All the protocols were approved by this committee (Permit Number: 2018BAD23B11).

## Author Contributions

XQ and ZL performed the majority of experiments. HL and TW participated the part of the experiments. XQ, JW, and YZ conceived the study and participated in its design and coordination. XQ prepared the manuscript. All authors have read and approved the final manuscript.

### Conflict of Interest

The authors declare that the research was conducted in the absence of any commercial or financial relationships that could be construed as a potential conflict of interest.
